# Absorption of PCB126 by upper airways impairs G protein-coupled receptor-mediated immune response

**DOI:** 10.1038/srep14917

**Published:** 2015-10-09

**Authors:** Ana Lúcia B. Shimada, Wesley S. Cruz, Rodrigo A. Loiola, Carine C. Drewes, Fabiane Dörr, Natália G. Figueiredo, Ernani Pinto, Sandra H. P. Farsky

**Affiliations:** 1 Department of Clinical and Toxicological Analyses, School of Pharmaceutical Sciences, University of São Paulo, São Paulo 05508-900 Brazil.

## Abstract

PCB126 is a dioxin-like polychlorinated biphenyl (PCB) environmental pollutant with a significant impact on human health, as it bioaccumulates and causes severe toxicity. PCB126-induced immune toxicity has been described, although the mechanisms have not been fully elucidated. In this study, an *in vivo* protocol of PCB126 intoxication into male Wistar rats by intranasal route was used, which has not yet been described. The intoxication was characterised by PCB126 accumulation in the lungs and liver, and enhanced aryl hydrocarbon receptor expression in the liver, lungs, kidneys, and adipose tissues. Moreover, an innate immune deficiency was characterised by impairment of adhesion receptors on blood leukocytes and by reduced blood neutrophil locomotion and oxidative burst activation elicited by *ex vivo* G protein-coupled receptor (GPCR) activation. Specificity of PCB126 actions on the GPCR pathway was shown by normal burst oxidative activation evoked by Toll-like receptor 4 and protein kinase C direct activation. Moreover, *in vivo* PCB180 intoxication did not alter adhesion receptors on blood leukocytes either blood neutrophil locomotion, and only partially reduced the GPCR-induced burst oxidative activation on neutrophils. Therefore, a novel mechanism of *in vivo* PCB126 toxicity is described which impairs a pivotal inflammatory pathway to the host defence against infections.

Polychlorinated biphenyls (PCBs) are lipophilic environmental contaminants called persistent organic pollutants (POPs), as they are resistant to environmental degradation and accumulate in the food chain. PCBs were widely used between 1930 and 1980 in industrial processes and products, like insulating fluids in electrical equipment, hydraulic systems, and pesticides^1^,^2^. Nowadays, PCB employment in many industrialised countries has a downward tendency due to a restriction on industry usage. Nevertheless, the uncontrolled disposal and storage of PCB residues and release in developing countries has contributed to environmental contamination and human intoxication^1^,^2^. Therefore, PCBs are found in high concentrations in the soil, water, and air in different parts of the world^2^,^3^. In addition, the presence of PCBs in building materials has contributed to indoor contamination, which has recently been considered an important and neglected pathway of exposure^4^,^5^,^6^,^7^. Absorptions by inhalation and by consumption of contaminated foods have provided elevated levels of PCBs in human samples, even in breast feed children, and PCBs intoxication lead to severe damage to the living organisms^1^,^2^,^8^,^9^.

Polyhalogenated aromatic hydrocarbons, such as 2,3,7,8-tetrachlorodibenzo-p-dioxin (TCDD), are agonists of the cytoplasmic aryl hydrocarbon receptor (AhR). By presenting AhR agonism, PCBs are called coplanar or dioxin-like PCBs, and PCB126 (3,3′,4,4′,5-pentachlorobiphenyl) is considered the main representative of this class. PCB126 toxicity is manifested by skin lesions, immune alterations, reproductive abnormalities, and increased risk of cardiovascular and liver diseases and diabetes^10^,^11^. The toxicity of dioxin-like PCBs on the immune system is controversial, and stimulation or depression of the system has been described. Furthermore, the mechanisms of toxic actions and the cross-talk between cell signalling pathways have not been completely elucidated^12^,^13^,^14^,^15^,^16^.

Leukocytes are bone-marrow-derived cells constantly delivered into the blood to maintain homeostasis and the immune host defence against injuries. Indeed, humoral and cytotoxic functions exerted by lymphocytes are fundamental to the acquired immune response; phagocytosis by neutrophils and monocytes are essential to host defence against microorganisms during the innate immune response. Leukocytes circulate from the blood to inflamed areas in response to chemotactic mediators activated in the plasma or released by resident cells, such as macrophages and mast cells, or by components of microorganisms. In this process, activated circulating leukocytes initially interact with endothelial cells from the vessel wall via the highly coordinated and sequential expression and activation of membrane adhesion molecules. In this context, leukocyte (L-selectin) and endothelial (P-selectin and E-selectin) selectins control the initial interaction of circulating leukocytes to the endothelium; leukocyte β2 integrins, endothelial intercellular (ICAM-1), endothelial vascular cell (VCAM-1), and leukocyte/endothelial platelet-endothelial (PECAM-1) cell adhesion molecules mediate the subsequent adhesion of leukocytes to the microvascular endothelium and diapedesis into inflamed tissues^17^,^18^. Subsequently, phagocytes crawl into the tissues and migrate into the inflamed area through a chemoattractant gradient in order to ingest and kill the microorganisms by releasing the contents of their granules and activating the oxidative burst^19^,^20^.

N-formyl-methionyl-leucyl-phenylalanine (fMLP) is part of the bacterial membrane and is also secreted by the mitochondria of eukaryotic cells during apoptosis^21^,^22^. It activates intracellular pathways responsible for leukocyte adhesion to the vessel wall, locomotion in the inflamed tissue, and killing. fMLP binds to the formyl peptide receptors 1 and 2 (FPR-1, FPR-2) found in the cell membrane, which belong to the superfamily of integral membrane protein receptors named seven-transmembrane domain G protein-coupled receptors (GPCRs). FPR1 presents high affinity towards fMLP, and activation of intracellular pathways through FPR2 is only evoked by elevated concentrations of the agonist. By affecting pivotal leukocyte functions, a balanced GPCR expression and activation on phagocyte membranes is fundamental to the host defence^23^. FPR1-deficient mice present increased inflammation and exacerbated liver injury during infection caused by bacterial lipopolysaccharide (LPS) injection^24^ and more severe inflammation and higher mortality in *pneumococcal meningitis* and *Listeria monocytogenes* infections^25^,^26^.

We designed an experimental study to show, for the first time, that *in vivo* PCB126 exposure modifies the pattern of adhesion and chemotactic receptors on circulating leukocytes, leading to impaired leukocyte adhesion, migration, and killing activities of neutrophils mediated by the GPCR pathway.

## Results

### Characterisation of *in vivo* PCB126 intoxication

**Figure 1:**
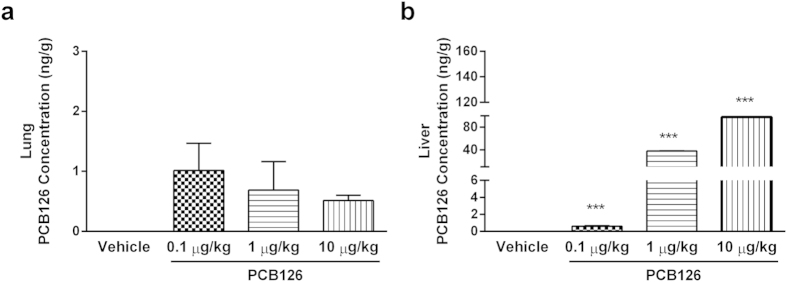
Detection of PCB126 in target tissues. PCB126 concentration (ng/g) in lung (**a**) and liver (**b**) tissues collected from male Wistar rats exposed to vehicle or PCB126 (0.1, 1, or 10 μg/kg once a day during 15 days). Five hours after the last exposures, samples were collected and PCB126 quantification was performed by GC/MS analysis. Data (n = 4 for each group) were analysed by one-way ANOVA followed by Tukey’s test. ***P < 0.001 *vs*. vehicle.

In order to determine whether pollutant exposure by nasal instillation would be detectable in absorptive and metabolic tissues, PCB126 concentrations in the lungs and liver were quantified by gas chromatography/mass spectrometry (GC/MS). The pollutant was detected in both tissues by the end of the PCB126 exposure, but was not found in tissues of vehicle-exposed rats used as controls (Fig. 1a,b).

**Figure 2:**
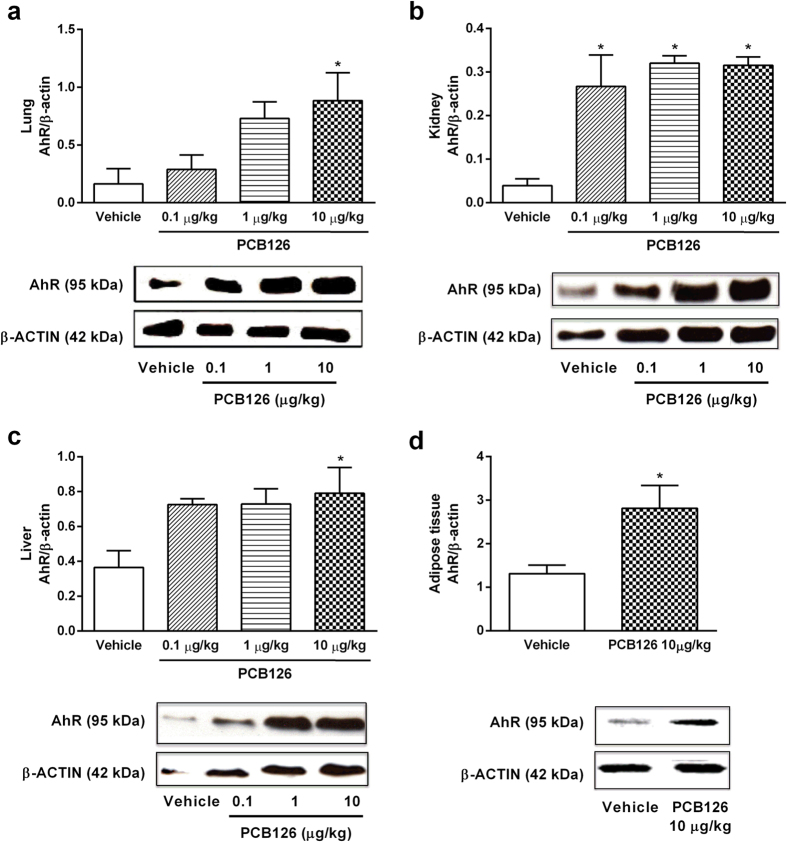
*In vivo* PCB126 exposure up-regulates AhR expression in target tissues. AhR protein expression in the lung (**a**), kidney (**b**) and liver (**c**) tissues collected from male Wistar rats exposed to vehicle or PCB126 (0.1, 1, or 10 μg/kg once a day during 15 days). AhR protein expression in the adipose tissue (**d**) collected from male Wistar rats exposed to vehicle or PCB126 (10 μg/kg once a day during 15 days). Five hours after the last exposures, samples were collected and AhR levels were quantified by Western blot. Data (n = 4 for each group) were analysed by one-way ANOVA followed by Tukey’s test (**a**–**c**) or Student’s *t*-test (**d**). *P < 0.05 *vs*. vehicle.

Moreover, we also detected the PCB126 exposure by measuring AhR expression, which has been described as a biological ligand of dioxin-like PCBs and PCBs exposure induces AhR expression^27^,^28^. Data presented in Fig. 2 show enhanced expression of the receptor in the lungs (Fig. 2a), kidneys (Fig. 2b), liver (Fig. 2c), and adipose tissue (Fig. 2d). Higher AhR expressions in the liver and kidneys were found after exposure to the lowest dose of PCB126 exposure (1 μg/kg).

### *In vivo* PCB126 exposure alters the expression of membrane adhesion molecule receptors on circulating leukocytes

**Figure 3:**
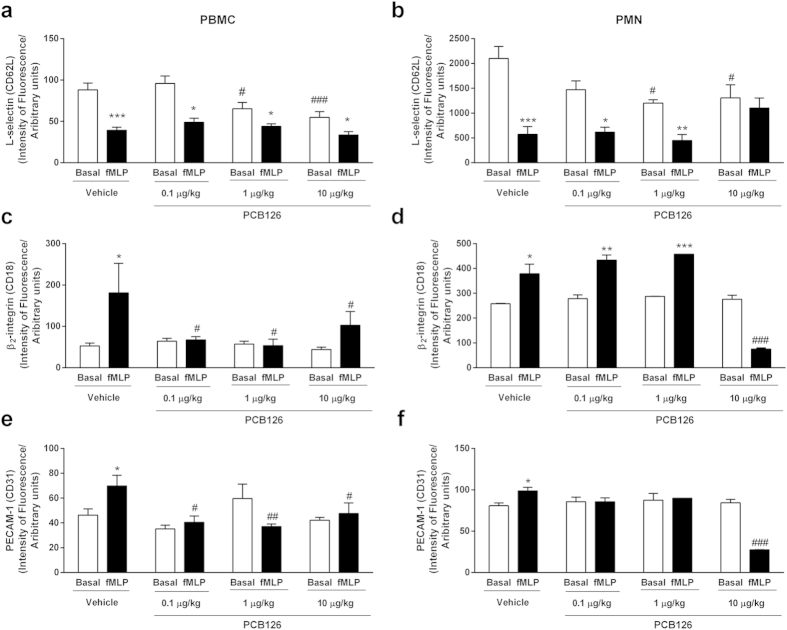
*In vivo* PCB126 exposure alters leukocyte adhesion molecule expression. Expression of L-selectin (**a**,**b**), β2-integrin (**c**,**d**), and PECAM-1 (**e**,**f**) in blood leukocytes collected from male Wistar rats exposed to vehicle or PCB126 (0.1, 1, or 10 μg/kg once a day during 15 days). Five hours after the last exposures, blood was collected and expressions of adhesion molecules were quantified in PBMC (**a**,**c**,**e**) or PMN (**b**,**d,f**) membranes by flow cytometry in basal conditions (white bars) or after fMLP (100 nmol/L for 1 hour at 37 °C, dark bars). Data (n = 5 for each group) were analysed by one-way ANOVA followed by Tukey’s test. *P < 0.05, **P < 0.01, and ***P < 0.001 *vs*. respective control (basal). ^#^P < 0.05, ^##^P < 0.01, and ^###^P < 0.001 *vs*. respective value in vehicle group.

Circulating leukocytes present a profile of membrane receptors able to promptly answer an aggressive stimulus. We found that PCB126 exposure reduced the normal expression of L-selectin on peripheral blood mononuclear (PBMC) and polymorphonuclear cells (PMN; [Fig. 3a,b, white bars]). No alteration was detected in β2 integrin or PECAM-1 adhesive molecules expression (Fig. 3c–f, white bars).

*In vitro* fMLP stimulation longer than 30 minutes causes cleavage of L-selectin on leukocyte membranes and subsequent expression of β2 integrin and PECAM-1^17^,^18^. This pattern of cell response was abolished in PMN and PBMC collected from PCB126-exposed rats and further *in vitro* activated by fMLP (Fig. 3a–f, dark bars).

**Figure 4:**
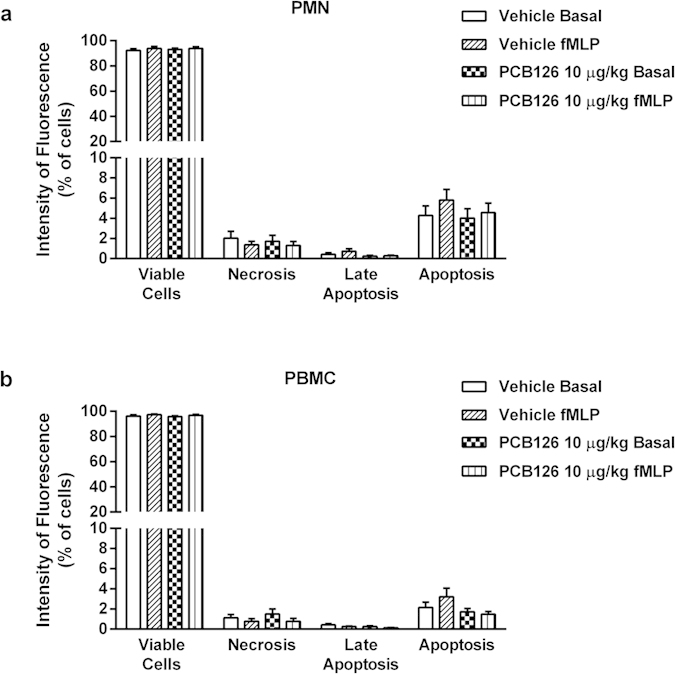
*In vivo* PCB126 exposure does not alter cell viability of blood leukocytes. Viability of PMN (**a**) and PBMC (**b**) cells collected from male Wistar rats exposed to vehicle or PCB126 (10 μg/kg once a day during 15 days). Five hours after the last exposures, circulating blood leukocyte was collected and cell death was evaluated by flow cytometry using anti-Annexin V and PI in basal conditions or after fMLP (100 nmol/L for 1 hour at 37 °C). Data (n = 5 for each group) were analysed by one-way ANOVA followed by Tukey’s test.

Moreover, these effects are not related to cytotoxic effects of PCB126 exposure. Even the higher dose of PCB126 did not cause PMN or PBMC apoptosis, necrosis, or late apoptosis under basal conditions or after *in vitro* fMLP exposure (Fig. 4).

**Figure 5:**
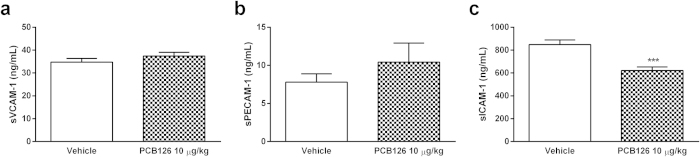
*In vivo* PCB126 exposure alters soluble levels of ICAM-1. Levels of sVCAM-1 (**a**), sPECAM-1 (**b**), and sICAM-1 (**c**) in serum collected from male Wistar rats exposed to vehicle or PCB126 (10 μg/kg once a day during 15 days). Five hours after the last exposures, blood from the abdominal aorta was collected and the levels of soluble adhesion molecules were evaluated in serum by ELISA. Data (n = 8 for each group) were analysed by Student’s *t*-test. ***P < 0.001 *vs*. vehicle.

Endothelial cells also express a pattern of adhesive molecules to maintain the homeostasis, which is modified following endothelial activation. Once expressed in non-activated endothelial cells, the majority of adhesion molecules are enzymatically cleaved and soluble forms are found in plasma after stimuli^29^. Levels of the soluble endothelial adhesion molecules VCAM-1 and PECAM-1 were not altered in serum from PCB126-exposed rats (Fig. 5a,b), but ICAM-1 levels were reduced after PCB126 exposure (Fig. 5c).

### *In vivo* PCB126 exposure modifies the neutrophil chemotaxis and oxidative stress induced by GPCR activation

**Figure 6:**
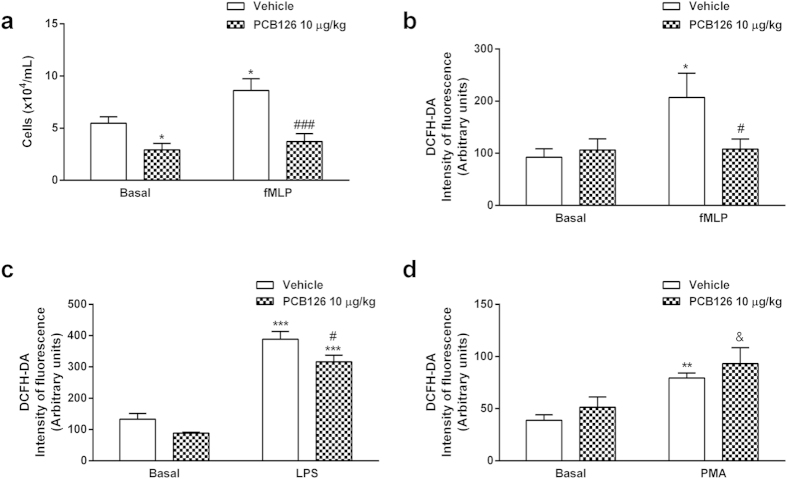
*In vivo* PCB126 exposure alters neutrophil chemotaxis and ROS production elicited by fMLP. Chemotaxis (**a**) and ROS production evoked by fMLP (**b**), LPS (**c**), or PMA (**d**) in neutrophils collected from male Wistar rats exposed to vehicle or PCB126 (10 μg/kg once a day during 15 days). Five hours after the last exposures, blood neutrophils were isolated from blood and chemotaxis (**a**) was evaluated using a Boyden Chamber in basal or fMLP (100 nmol/L) conditions. ROS production induced by fMLP (100 nmol/L) (**b**), LPS (5 μg/mL) (**c**), or PMA (100 ng/mL) (**d**) were measured by flow cytometry. Data (n = 8 for each group) were analysed by one-way ANOVA followed by Tukey’s test. *P < 0.05,**P < 0.01 and ***P < 0.001 *vs*. values in vehicle treated rats; ^#^P < 0.05, ^###^P < 0.001 *vs*. vehicle fMLP; ^&^P < 0.05 *vs*. PCB126 10 μg/kg in basal condition.

**Figure 7:**
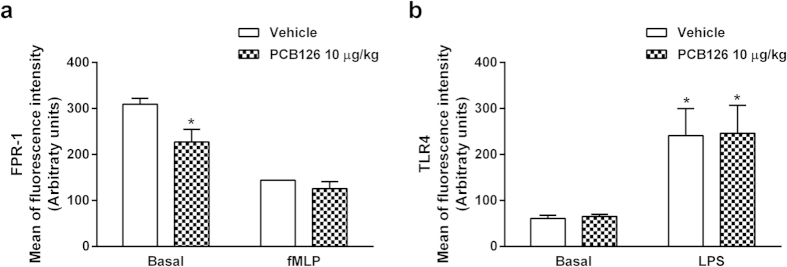
*In vivo* PCB126 exposure alters FPR-1 expression in blood neutrophils. FPR-1 (**a**) and TLR4 expression (**b**) in blood neutrophil collected from male Wistar rats exposed to vehicle or PCB126. Male Wistar rats were exposed to vehicle or PCB126 (10 μg/kg once a day during 15 days). Five hours after the last exposures, blood neutrophils were isolated and stimulated by fMLP (100 nmol/L) or LPS (5 μg/mL). Quantification of FPR-1 (**a**) and TLR4 (**b**) membrane expression was performed by flow cytometry. Data (n = 6 for each group) were analysed by one-way ANOVA followed by Tukey’s test. *P < 0.05 *vs*. respective basal.

In order to confirm the impact of PCB126 on leukocyte functions, we investigated typical activities of GPCR activation by fMLP on circulating neutrophils. Blood neutrophils collected from PCB126-exposed animals presented diminished chemotactic profiles under basal conditions or after fMLP stimulation (Fig. 6a) and reduced reactive oxygen species (ROS) production after fMLP stimulation (Fig. 6b). On the other hand, ROS production evoked by LPS (Fig. 6c) or phorbol-12-myristate-13-acetate (PMA; Fig. 6d) stimulations were not impaired in neutrophils obtained from PCB126-exposed rats. Together, these data suggest that *in vivo* PCB126 exposure primarily affects the GPCR pathway activated by fMLP. Moreover, PCB126 exposure reduced the basal expression of FPR1, the GPCR binding to fMLP, however, did not alter the basal expression of Toll-like receptor 4 (TLR4), the receptor to LPS or even its expression after LPS stimulation (Fig. 7).

### *In vivo* PCB180 exposure only reduces GPCR-induced oxidative burst activation in PMN cells

**Figure 8:**
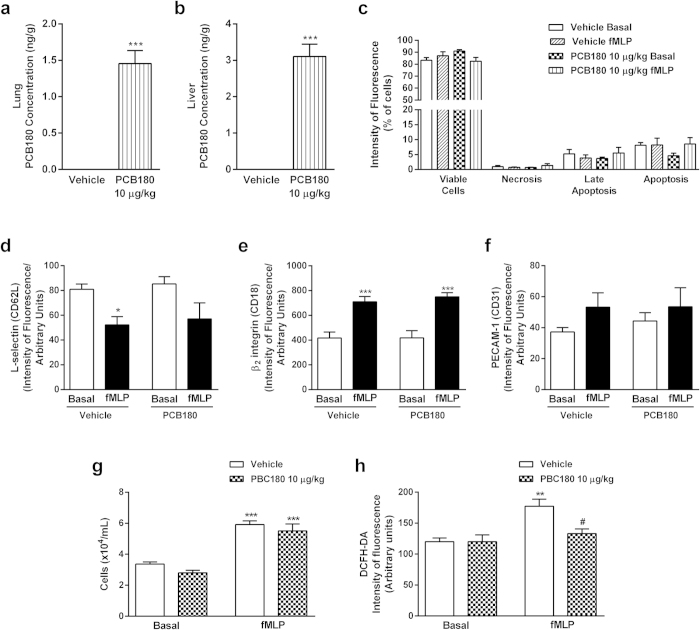
*In vivo* PCB180 exposure only alters neutrophil ROS production elicited by fMLP. Male Wistar rats were exposed to vehicle or PCB180 (10 μg/kg, once a day during 15 days). Five hours after the last exposures, samples were collected. Blood PMNs were treated with vehicle or fMLP (100 nmol/L). PCB180 concentration (ng/g) in lung (**a**) and liver (**b**) tissues, viability of blood PMN (**c**), expression of L-selectin (**d**), β2-integrin (**e**), and PECAM-1 (**f**) in blood PMN, blood PMN chemotaxis (**g**) and PMN ROS production (**h**). Data (n = 6–8 for each group) were analysed by one-way ANOVA followed by Tukey’s test. *P < 0.05, **P < 0.01 and ***P < 0.001 vs respective basal or vehicle; ^#^P < 0.05 vs Vehicle + fMLP.

*In vivo* PCB180 exposure was carried out to point out the effects of non-coplanar PCB on GPCR activation on leukocytes. Data obtained detected levels of PCB180 in the lungs and liver (Fig. 8a,b), showing the absorption by intranasal route. The *in vivo* exposure did not alter the viability of circulating PMN (Fig. 8c), either basal expression of adhesion molecules L-selectin, β2 integrin or PECAM-1 in PMN (Fig. 8d–f). Furthermore, modifications on adhesion molecules induced by *in vitro* fMLP stimulation were equivalent on PMN collected from vehicle or PCB180 exposed rats (Fig. 8d–f). Adhesion molecules expressions and cell viability were similar on PBMC isolated from vehicle or PCB180 exposed animals (data not shown). Moreover, *in vivo* PCB180 exposure did not alter the neutrophil chemotactic response to fMLP (Fig. 8g), nevertheless reduced the fMLP-induced oxidative burst activation (Fig. 8h).

## Discussion

The deleterious effects of environmental pollutants on human health are incontestable. Thus, understanding their toxic mechanisms of action in order to determine earlier, selective, and sensible biological endpoints has been a challenge to the toxicology^30^,^31^. In this context, we designed a set of experiments to investigate the toxic mechanisms of inhaled PCB126 on immune system, and data obtained point out the harmful actions of *in vivo* PCB126 exposure on blood cell, which impairs the host defence mediated by GPCR activation.

Based on the dispersal of PCBs in the environment, we investigated PCB126 toxicity through by the intranasal route, which has not been previously studied. PCB126 levels detected in lung and liver samples, and higher expression of AhR in several tissues, such as the liver, lungs, kidneys, and adipose tissue, also validated the pathway of administration.

Mature blood leukocytes present a distinct phenotype to maintain homeostasis, and a portfolio of membrane receptors must be expressed in order to prompt host immune defences to aggression. In this context, we detected that *in vivo* PCB126 exposure reduces the constitutive expression of L-selectin on PMN and PBMC membranes. L-selectin mediates lymphocyte binding to high endothelial venules in the recirculation process^32^,^33^ as well as the initial interactions of phagocytes onto endothelial microvessels during an innate response^17^. Furthermore, L-selectin-activated signalling leads to the subsequent adhesive cascade of events between leukocytes and endothelium and progression of an inflammatory response^32^,^33^. Based on reduced L-selectin expression on cell membranes, it could be supposed that blood leukocytes from PCB126-exposed rats would not properly respond to a subsequent activation. fMLP was the chosen stimulating agent to answer this question due to the relevance of the GPCR pathway for the infectious host defence^23^,^24^,^25^,^26^. Binding of fMLP to FPR-1 induces cleavage of L-selectin by membrane metalloproteases and subsequent expression of the adhesive β2 integrin and PECAM-1 receptors on phagocyte membranes^34^. Indeed, fMLP-induced L-selectin enzymatic shedding and β2 integrin and PECAM-1 expressions were impaired in PBMC and PMN membranes from PCB126-exposed rats.

It was previously shown that *in vitro* incubation of rat neutrophils simultaneously with PCB126 and fMLP caused cytotoxicity^35^. Therefore, the possibility that *in vivo* PCB126 exposure could lead to neutrophil death after *in vitro* fMLP stimulation was excluded by virtue of the absence of apoptosis or necrosis in the cell suspensions.

No evidence regarding the effects of PCBs on leukocyte adhesion molecule expression and activity have been previously shown; nevertheless, *in vitro* exposures of PCBs to different cultured endothelial cells have shown up or down expression of immunoglobulins or selectin molecules^36^,^37^,^38^. Our data do not support the *in vitro* evidences, as soluble levels of endothelial adhesion molecules were not detectable in sera of PCB126-exposed rats. It is worth mentioning that detection of soluble adhesion molecules is a reliable method to determine endothelial cell activation, as cell membrane expressed molecules are enzymatically cleaved, resulting in elevated levels of soluble fragments in blood^29^,^39^,^40^.

**Figure 9:**
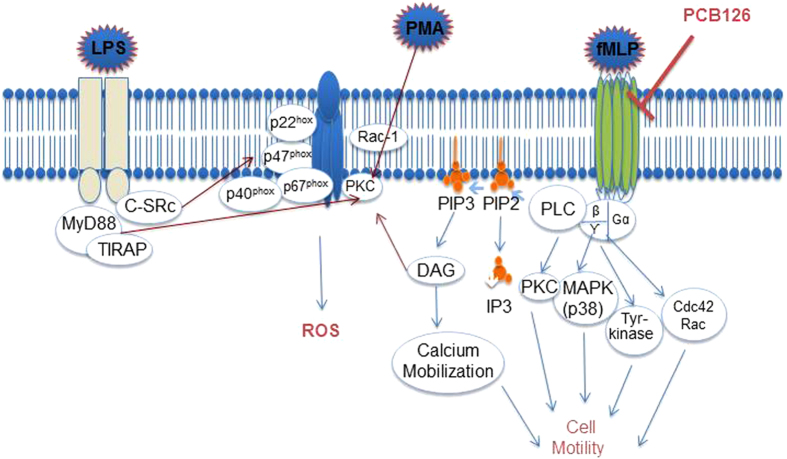
PCB126 actions on LPS-, PMA-, or fMLP-induced ROS production and fMLP-induced chemotaxis. LPS: lipopolysaccharides; PMA: phorbol-12-myristate-13-acetate; fMLP: N-formyl-methionyl-leucyl-phenylalanine; MYD88: myeloid differentiation primary response gene 88; TIRAP: Toll-interleukin 1 receptor (TIR) domain containing adaptor protein; PKC: protein kinase C; DAG: diacyl-glycerol; RAC-1: Ras-related C3 botulinum toxin substrate 1; PIP3: phosphatidylinositol (3,4,5)-triphosphate; PIP2: phosphatidylinositol 4,5-bisphosphate; PLC: phospholipase C; MAPK: mitogen-activated protein kinase; Tyr-Kinase: tyrosine kinase; CDC42 Rac: cell division control protein 42 homolog; C-Src: tyrosine-protein kinase CSK.

Neutrophils are highly responsive to fMLP; hence, circulating neutrophils were employed to further confirm the actions of *in vivo* PCB126 exposure on the GPCR pathway. By binding to FPR-1 on neutrophils, fMLP activates membrane diacylglycerol and phospholipase C, which leads to intracellular activation of phosphatidylinositol 3-kinase and other phosphokinases isoforms along with calcium mobilisation. This mechanism leads to reorganisation of microtubules and microfilament to hammer out focal points to adhesion and chemotaxis on the extravascular matrix^22^. Moreover, kinase activation causes NF-κB translocation into the nucleus, with consequent expression of inflammatory proteins, such as cell adhesion molecules and cytokines, and also causes activation of p47phox and NADPH oxidase to trigger the oxidative burst^22^. Interference of PCB126 in the GPCR pathway was shown by reduced chemotaxis and oxidative burst activation in response to fMLP and, conversely, normal oxidative burst activation in response to PMA, a direct activator of the intracellular protein kinase C, or to LPS, which binds to the TLR-4 and activates MyD88/TRAM intracellular cascade. Moreover, neutrophils obtained from PCB126-exposed rats presented reduced FPR-1 expression on the membrane, but normal TLR4 expression. Oxidative burst activation was the chosen endpoint to detect the mechanism of PCB126 intoxication on neutrophils, as it is induced by the three agents we employed by different pathways, as shown in Fig. 9.

Based on the data obtained, we conclude that PCB126 exposure does not affect the NAPDH oxidase complex activated by kinases but impairs the intracellular cascade induced by GPCR activation, as proposed in Fig. 9. Moreover, equivalent FPR-1 internalisation after fMLP binding was detected in neutrophils obtained from vehicle- or PCB126-exposed rats. Therefore, GPCR desensitisation, G protein coupling, and phosphorylation pathways may also be directly impaired by *in vivo* PCB126 exposure. These hypotheses will be further investigated.

It is worth mentioning that similar i*n vivo* exposure to PCB180 did not modify the basal pattern of adhesion molecule expression on leukocytes, either did not impair the leukocyte adhesion molecule expression or neutrophil chemotaxis induced by GPCR activation. Nevertheless, the PCB180 exposure reduced the ability of neutrophils to activate the oxidative burst, suggesting an effect of PCB180 *in vivo* exposure on activation of p47phox and NADPH oxidase. Therefore, our data show that dioxin and non-dioxin PCB differently modulate immune functions of blood leukocytes.

The complexity of the immune system creates a huge number of targets to xenobiotics, and the consequent correlation of environmental pollution and infectious diseases has been fully shown^41^,^42^,^43^. Considering that innate immune cells are the first barrier to aggression, are responsible for antigen presentation, and act as connectors of innate and acquired responses^19^,^20^,^44^, the novel mechanism demonstrated here to operate in PCB126 exposure, which impairs GPCR pathway activation in innate immune cells, may be an early toxic effect leading to a deficient host defence due to dioxin-like PCB intoxications.

## Material and Methods

### Animals

Male Wistar rats (180–220 g) were purchased from the Animal Facility of the School of Pharmaceutical Sciences and Chemistry Institute at the University of Sao Paulo. The animals were fed a standard pellet diet and water *ad libitum*, and, before each experimental procedure, they were anaesthetised with ketamine/xylazine solution (80:8 mg/kg, respectively, intraperitoneally [i.p.]). All procedures were performed according to the guidelines of the Brazilian Society for the Science of Laboratory Animals (SBCAL) and approved by the Institutional Animal Care and Use Committee (Protocol number 315).

### Protocol of *in vivo* PCB126 or PCB180 exposure

Once a day over the course of 15 days, the animals were exposed to PCB126 at 0.1, 1, or 10 μg/kg of body weight. Others groups of animals were exposed to PCB180 at 10 μg/kg of body weight. Control animals received only the vehicle solution of PCB126 or PCB180 that consisted of a 0.5% dimethyl sulfoxide (DMSO) saline solution. The exposure was done by nasal instillation (50 μL), and 5 hours after the last PCB126, PCB180 or vehicle exposure, the animals were anaesthetised and euthanised, and samples were collected. PCB126 (purity 99%) was purchased from AccuStandard (New Haven, CT, USA) and PCB180 (purity 99%) was purchased from Sigma-Aldrich (St Louis, USA). Schedule of PCB126 exposure was set up after previous dose/effect experiments, and the lower dose was chosen because it did not cause blood cell death but elevated the levels of PCB126 and AhR in the tissues of the lungs and liver (10 μg/kg). PCB180 dose was based on PCB126 dose, which caused alterations on GPCR pathways on leukocytes.

### AhR expression: Western blotting assay

After *in vivo* PCB126 exposure, equal amounts of protein (100 μg) from liver, lung, kidney, or adipose tissue lysate were separated on 15% sodium dodecyl sulphate-polyacrylamide gels (Thermo Scientific, Waltham, MA, USA) for 150 minutes at 100 V and transferred onto a 0.2 μm polyvinylidene fluoride (PVDF) membrane (Thermo Scientific, Waltham, MA, USA) at 100 V for 90 minutes. The membranes were blocked for 1 hour at room temperature with 5% non-fat milk and washed six times with Tris-buffered saline plus 0.5% Tween^®^20 (TBST) buffer for 10 minutes each time. The membranes were then incubated overnight at 4 °C with a specific primary antibody against AhR (1:1.000 dilution, vol/vol). Then, the membranes were washed six times with TBST buffer, incubated for 45 minutes with goat anti-mouse-IgG horseradish peroxidase (HRP; 1:30.000 dilution, vol/vol), and washed six times with TBST. The immune reactive bands were visualised with an electrochemiluminescence (ECL) system (Thermo Scientific, Waltham, MA, USA). The β-actin expression was used as an internal loading control for protein expression normalisation. All antibodies were obtained from Abcam (Cambridge, MA, USA).

### PCB126 and PCB180 quantification in biological samples by gas chromatography/mass spectrometry (GC/MS) analysis

Analyses of the PCB126 and PCB180 levels in lung and liver tissues from the *in vivo* exposed rats were performed as described by Poli *et al.* (2009)^45^. Briefly, 500 mg of lung or liver tissue were homogenised in an aqueous 10% KCl solution and spiked with PCB52 as an internal standard (IS; AccuStandard, New Haven, CT, USA). Samples were incubated at 100 °C and extracted by solid phase micro-extraction (SPME) on a polyacrylate fibre (85 μm, Supelco, Bellefonte, PA, USA) in headspace mode (HS-SPME) for 40 minutes by a GC Sampler 80 (Agilent Technologies, Palo Alto, CA, USA), which was used to turn the samples to a gas phase. Detection was performed using a 7890A gas chromatograph coupled with a 5975C mass spectrometer (Agilent Technologies, Palo Alto, CA, USA), and the analytes were separated on a HP-5MS column (30 m × 0.25 mm ID; 0.25 μm film, Agilent Technologies, Palo Alto, CA, USA) using helium as a gas carrier at a flow-rate of 1 mL/min. The injection port was set at 280 °C (in splitless mode) and the oven temperature programme was 30 °C/min from 100 to 280 °C (hold for 5 minutes). The transfer line and MS source temperature were maintained at 280 °C. The MS was operated in electron impact (EI) ionisation mode at 70 eV. The acquisition of the ions *m/z* 220, 222, 290, 292, and 294 (at 5.52 minutes for IS) and *m/z* 326 and 328 (at 7.05 minutes for PCB126); and *m/z* 394, 396 and 398 (at 8.02 minutes, for PCB180) was made in selected ion monitoring (SIM) mode.

### Cytotoxicity, adhesion molecules, membrane receptor expression, and oxidative burst by flow cytometry

Cells collected from the abdominal aorta of vehicle, PCB126 or PCB180-exposed rats were used to quantify cell adhesion expression, cytotoxicity, membrane receptor expression, and reactive oxygen species (ROS) generation. Briefly, erythrocytes were lysed by washes with NaCl 0.2 and 1.6% to the samples, and total leukocytes were recovered after washing the resulting pellet with Hank’s balanced salt solution (HBSS). Total leukocytes were incubated with HBSS (basal) or fMLP (100 nmol/L for 1 hour at 37 °C). Thereafter, leukocytes were incubated for 20 minutes in the dark at 4 °C with 100 μL of a 1:100 dilution (vol/vol) of rat monoclonal antibody (L-selectin or β2 integrin fluorescein-isothiocyante [FITC]-conjugated or PECAM-1 phycoerytrin [PE]-conjugated to quantify the expression of membrane adhesion molecules. In another group of samples, leukocytes were incubated with anti-anexin-V (FITC-conjugated; 1:100 vol/vol for 20 minutes at 4 °C) and propidium iodide (PI, immediately before flow cytometry analysis) to quantify cell viability. The PMN and PBMC cell subpopulations were recognised by FSC (forward scatter) and SSC (side scatter) parameters, which evaluate the size and internal complexity of cells, respectively.

In addition, blood neutrophils from vehicle or PCB126-exposed animals were isolated using a Percoll gradient (Sigma-Aldrich, St. Louis, MO, USA) and were stimulated with fMLP (100 mmol/L for 1 hour at 37 °C) or LPS (5 μg/mL for 1 hour at 37 °C). Afterwards, they were incubated with anti-FPR-1 or anti-TLR4 antibodies (FITC- or PE-conjugated, respectively, 1:100 vol/vol for 20 minutes at 4 °C) and analysed by flow cytometry FACSCanto II® (Becton & Dickinson; San Jose, CA, USA).

Furthermore, the fluorescent dye 2′,7′-Dichlorodihydrofluorescin diacetate (DCFH-DA, 340 μmol/L; diluted in PBS) was added to 2 × 10^5^ neutrophils obtained from vehicle or PCB126 -exposed rats in a final volume of 1.1 mL to measure the intracellular ROS^46^. Cells were maintained at 37 °C for 30 minutes and rinsed with EDTA (3 mmol/L; 2 mL) to remove excess dye. Cells were incubated with an inflammatory stimuli, such as fMLP (100 nmol/L for 10 minutes at 37 °C), lipopolysaccharide from *E. coli* 026:B6 (LPS; 5 μg/mL for 30 minutes at 37 °C), or phorbol-12-myristate-13-acetate (PMA; 100 ng/mL for 15 minutes at 37 °C). Blood neutrophils were collected from PCB180 exposed rats as described above and incubated with fMLP (100 nmol/L for 10 minutes at 37 °C) for quantification of intracellular ROS^46^.

Cells were acquired in a FACSCanto II® flow cytometer (Becton & Dickinson, San Jose, CA, USA). Data from 10,000 events were obtained, and only morphologically viable cells were considered for analysis. Results are presented as arbitrary units of fluorescence. The anti-FPR-1-FITC antibody was acquired from Biorbyt Ltd. (Cowley Road, Cambridge, UK) and the additional antibodies were purchased from BD Pharmingen (San Diego, CA, USA). fMLP, LPS, PMA, and DCFH-DA were obtained from Sigma-Aldrich (St. Louis, MO, USA).

### Soluble adhesion molecules by ELISA

After the last exposures, blood was collected from the abdominal aorta and serum isolated by centrifugation (1,500 *g* for 10 minutes at 4 °C), frozen, and stored at −80 °C until analysis. The amount of rat-soluble adhesion molecules, particularly ICAM-1, VCAM-1, and PECAM-1, was immunoenzymatically determined using commercial kits from MyBioSource (San Diego, CA, USA).

### *In vitro* chemotaxis

Blood neutrophils isolated from vehicle, PCB126 or PCB180-exposed rats, as described above, were loaded on the top wells (25 μL, 1 × 10^5^ cells) of a commercially available Neuroprobe ChemoTx^®^-111-8™ 96-well plate equipped with a 8 μm pore size filter and 3.2 mm diameter (Neuroprobe, Leamington Spa, UK). As a chemoattractant, 300 μL of 100 nmol/L fMLP were added in the bottom wells and the plate was incubated for 2 hours (at 37 °C, 5% CO_2_). Migrated neutrophils were counted in a Neubauer haemocytometer after dilution in Turk’s solution.

### Statistical analysis

Data are expressed as mean ± standard error of the mean (SEM). The comparison between two groups was analysed by Student’s *t*-test, while comparisons between three or more groups were analysed by one-way ANOVA followed by Tukey’s test. All statistical analyses were performed by using the GraphPad Prism 5.0 software (San Diego, CA, USA), and a value of P < 0.05 was considered significant.

## Additional Information

**How to cite this article**: Shimada, A. L. B. *et al.* Absorption of PCB126 by upper airways impairs G protein-coupled receptor-mediated immune response. *Sci. Rep.* **5**, 14917; doi: 10.1038/srep14917 (2015).
